# Acclimation to a thermoneutral environment abolishes age-associated alterations in heart rate and heart rate variability in conscious, unrestrained mice

**DOI:** 10.1007/s11357-019-00126-7

**Published:** 2019-11-27

**Authors:** Jessie E. Axsom, Alay P. Nanavati, Carolyn A. Rutishauser, Janet E. Bonin, Jack M. Moen, Edward G. Lakatta

**Affiliations:** 1 Intramural Research Program, Laboratory of Cardiovascular Science, National Institute on Aging, National Institutes of Health, 251 Bayview Boulevard, Suite 100, Baltimore, MD 21224 USA; 2 School of Nursing, University of Pennsylvania, Philadelphia, PA USA; 3 College of Osteopathic Medicine, University of New England, Biddeford, ME USA; 4 Department of Anatomy, Case Western Reserve University, Cleveland, OH USA; 5 School of Medicine, University of Maryland, Baltimore, MD USA; 6 Cellular and Molecular Physiology, Yale University, New Haven, CT USA

**Keywords:** Thermoneutrality, Aging, Heart rate, Heart rate variability, Cardiac autonomic modulation

## Abstract

Mice are among the most widely used translational models of cardiovascular aging and offer a method to quickly assess lifespan changes in a controlled environment. The standard laboratory temperature (20–22 °C), however, imposes a cold stress on mice that causes an increase in sympathetic nervous system–mediated activation of brown adipose tissue (BAT) to maintain a core body temperature of 36–37 °C. Thus, while physiologic data obtained recapitulate human physiology to a certain degree, interpretations of previous research in mice may have been contaminated by a cold stress, due to housing mice below their thermoneutral zone (30 °C). The purpose of this investigation was to examine how chronic sympathetic stimulation evoked by acclimation to 20 °C might obscure interpretation of changes in autonomic modulation of heart rate (HR) and heart rate variability (HRV) that accompany advancing age. HR and HRV before and after administration of a dual-autonomic blockade were measured via in-vivo ECG in young (3 months) and aged (30 months) male C57BL/6 telemetry-implanted mice following temperature acclimation for 3 days at 30 °C or 20 °C. Mean basal and intrinsic HR of both young and aged mice became markedly reduced at 30 °C compared to 20 °C. In both age groups, HRV parameters in time, frequency, and non-linear domains displayed increased variability at 30 °C compared to 20 °C under basal conditions. Importantly, age-associated declines in HRV observed at 20 °C were ameliorated when mice were studied at their thermoneutral ambient temperature of 30 °C. Thus, an accurate understanding of autonomic modulation of cardiovascular functions in mice of advanced age requires that they are housed in a metabolically neutral environment.

**Electronic supplementary material:**

The online version of this article (10.1007/s11357-019-00126-7) contains supplementary material, which is available to authorized users.

## Introduction

The autonomic nervous system (ANS) innervates the heart through both sympathetic input via sympathetic nerves and parasympathetic input via the vagus nerve to cardiac neuronal ganglia (Shaffer et al. 2014). The heart’s beating rate (HR) and inter-beat variability (heart rate variability, HRV) are modulated by complex heart-brain-heart interactions involving both the ANS and intrinsic sinoatrial node (SAN) pacemaker cell functions (Shaffer et al. 2014; Kember et al. 2011).

Ultradian short-term HRV is characterized by an array of parameters that relate to the variability of the inter-beat intervals linked to respiratory cycles and are grouped into three categories: time domain parameters that describe interval variability in an electrocardiogram (ECG) using calculated statistics of RR interval time; frequency domain parameters that sort out rhythms that are buried within the ECG time-series; and non-linear domain parameters that describe variation in fractal-like behavior of intervals within the ECG time-series (Task Force of the European Society of Cardiology and the North American Society of Pacing and Electrophysiology 1996; Costa et al. 2008).

Heart rhythm patterns can range from coherent to complex. Coherency within complex physiological systems can be described as the degree of synchronization among oscillators intrinsic to a complex system (McCraty and Shaffer 2015). A coherent rhythm has low beat-to-beat variability and is associated with a high sympathetic input to the SAN, or a high level of adenylyl cyclase signaling intrinsic to SAN cells (Moen et al. 2019). More complex rhythms have a higher beat-to-beat variability, conferred by a high parasympathetic input or lower intrinsic SAN cell adenylyl cyclase activity (Thayer et al. 2010; Moen et al. 2019) and are considered to be indicative of a more balanced rhythm, reflecting heart homeostatic adaptations to the body’s physiologic rhythms, e.g., breathing (Shaffer et al. 2014); more coherent heart rhythms, on the other hand, are associated with a myriad of poor health outcomes, such as increased mortality following myocardial infarction (MI) or post-traumatic stress disorder (PTSD) (Bigger Jr et al. 1992a, b, 1993; Cohen et al. 2000; Kleiger et al. 1987; La Rovere et al. 1998; Thayer et al. 2010; Thayer et al. 2010)

HR, HRV, and ANS modulation of these are all profoundly affected by age, even in the absence of disease, consistent with the idea that aging itself is a disease (Lakatta 2015). Maximum HR in humans has been found to linearly decrease with advancing age, and is independent of physical fitness (Tanaka et al. 2001; Londeree and Moeschberger 1984; Christou and Seals 2008; Higginbotham et al. 1986). Intrinsic HR, i.e., HR in the presence of double autonomic blockade and relatively free from ANS influence, has also been found to decrease with advancing age, although the oldest subjects were of middle age (Jose and Collison 1970; Christou and Seals 2008). Age-associated changes in HR are accompanied by a decline in HRV (De Meersman and Stein 2007; Umetani et al. 1998; Antelmi et al. 2004; Lipsitz and Goldberger 1992). Changes in responses of cardiovascular tissues to autonomic receptor stimulation that occur during aging are partially accountable for the age-associated decline in HRV. Both adrenergic and muscarinic receptor responses to neurotransmitters become blunted with increasing age (Fleg et al. 1985; White and Leenen 1994). This, in part, underlies a reduced baroreceptor sensitivity as age increases. There is also an age-associated loss of beta-adrenergic receptor (BAR)–induced vasorelaxation (Pan et al. 1986; Schutzer et al. 2011) and evidence of decreased postsynaptic efficiency (Fleg and Strait 2012). But the age-associated decline in HRV not only results from a reduction in the effectiveness of postsynaptic autonomic receptor responses of pacemaker cells residing in SAN tissue but also to deterioration of intrinsic pacemaker clock functions of SAN cells (Yaniv et al. 2014; Liu et al. 2014; Moen et al. 2019).

Age-associated changes in HR or HRV in humans are recapitulated in animal models, of which small rodents provide valuable mechanistic insight into HR and HRV declines with aging. There is evidence that age-associated changes in ion channel gene expression contribute to SAN dysfunction in rats (Tellez et al. 2011). This was corroborated by evidence that changes in ion channel expression are associated with decreased SAN cell excitability and decreased intrinsic HR in aged mice (Larson et al. 2013). In addition to changes in SAN cell ion channels, Ca^2+^ signaling mechanisms within SAN cells are also dysfunctional in aging mice (Liu et al. 2014). Thus, reduced intrinsic SAN function, as well as a reduced SAN cell response to autonomic input, could lead to a loss of complexity in the HRV of aged mice (Yaniv et al. 2016).

Although mice serve as a valuable cardiovascular translational model, one of the oft-cited criticisms is that mice have an apparent sympathetically dominated heart rate at rest (Yaniv et al. 2016). However, an ambient laboratory temperature (LT) of 20–22 °C, at which mice are typically maintained, is well below their metabolic thermoneutral zone (TN) of 29–30 °C (Gordon et al. 1998; Gordon 2012; Lodhi and Semenkovich 2009; Fischer et al. 2018), i.e., the temperature at which core body temperature (36–37 °C) is maintained with the least energy expenditure (Ravussin et al. 2012). Due to a large surface area to body mass ratio, mice at LT employ non-shivering thermogenesis via sympathetically-driven stimulation of brown adipose tissue to maintain their core body temperature at LT (Himms-Hagan 1985; Kawate et al. 1994; Swoap et al. 2008; Swoap et al. 2004). This chronic sympathetic stimulation, however, has dramatic effects on whole body physiology, including metabolic and cardiovascular system function (Feldmann et al. 2009; Williams et al. 2003; Swoap et al. 2008; Swoap et al. 2004).

But when mice are housed at their TN, a marked reduction in heart rate occurs and a formidable vagal component of their heart rate regulation is revealed (Swoap et al. 2008). Thus, the vast majority of research in mice to date has been by far conducted on cold-stressed mice and therefore age-associated changes that have been identified in HR, HRV, and other aspects of ANS function and cardiovascular health in mice might have been distorted by the cold-induced sympathetic drive. We hypothesized that reductions in HR, loss of complexity, and an increase in coherency of heart rhythm in mice as they age (Yaniv et al. 2016; Larson et al. 2013) may largely be attributable to the ambient LT (20–22 °C) at which the mice were acclimated. To this end, we analyzed ECG time series from young (3–4 months, *n* = 14) and old (30 months, *n* = 17) telemetry-implanted untethered conscious mice in the absence and presence of dual autonomic blockade following acclimation to ambient temperatures of 20 °C or 30 °C. Our results confirm the hypothesis that cold-stress of a lower ambient temperature (20 °C) produces overdrive sympathetic stimulation that elevates HR and reduces HRV, leading to distorted perspectives on age-associated changes in HR and its rhythm. Importantly, our results are the first to not only provide in-depth analysis of mouse HRV response to ambient temperature but also show for the first time, that acclimation to a thermoneutral ambient temperature (30 °C) reverses age-associated declines in HR and HRV at standard laboratory ambient temperature (20 °C).

## Methods and materials

### In-vivo data collection

Studies were implemented in compliance with the Guide for the Care and Use of Laboratory Animals by the National Institutes of Health. The animal study protocol was approved by the Animal Care and Use committee of the National Institute on Aging (ASP 471-LCS-2019). Mice were kept on a standard 12-h light-dark cycle, single-housed with corn pop bedding, and fed standard chow ad-libitum. Young (3–4 months of age, *n* = 14) and old (28–30 months of age, *n* = 17) male C57/BL6 mice were implanted with telemetry devices (ETA-F10, Data Sciences International, St. Paul, MN). After a 2-week recovery period, mice were acclimated to 20 °C for 3 days in a temperature-controlled Comprehensive Laboratory Animal Monitoring System (CLAMS, Columbus Instruments). On day 4, a 1.5-h baseline ECG at a sampling rate of 1 KHz was recorded in the CLAMS. A double autonomic blockade consisting of atropine (0.5 mg kg^−1^) and propranolol (1 mg kg^−1^) diluted in saline (6.6 mL kg^−1^) were administered via an intraperitoneal (i.p.) injection, and the ECG recording continued for another 1.5 h. The maximum response to double autonomic blockade typically occurred within 30 min of administration but the recording was continued to ensure complete capture of response. Following a 48-h wash-out period, mice were acclimated to a 30 °C environment for 3 days. The double autonomic blockade ECG protocol was then repeated then at 30 °C.

### ECG time-series analysis

ECG time-series were analyzed using Labchart software (version 7.3.7), and HRV was calculated using custom-built python 3.5 software as previously described in detail by Moen et al. (2019). Because of marked heart rate responses to ambient temperatures, cut-off parameters for frequency domain HRV categories were adjusted for changes in the beating rate (Behar et al. 2018): high frequency power spectral density (HF.PSD) was defined as 1.50–5.0 Hz at 20 °C and 0.75–3.0 Hz at 30 °C; low frequency power spectral density (LF.PSD) was defined as 0.5–1.5 Hz at 20 °C and 0.2–0.75 Hz at 30 °C; very low frequency power spectral density (VLF.PSD) was defined as 0–0.5 Hz at 20 °C and 0–0.2 Hz at 30 °C. Core body temperature data was also extracted from the telemetry devices.

### Statistical analysis

Statistical analysis was completed using RStudio and R3.6.1. Data are reported as means (standard error). Linear mixed effects models were employed to discover age, temperature, and drug effects and interactions among these effects (lmerTest, Kuznetsova et al. 2016). Linear mixed effects models account for repeated measures on the same animals and uneven group sizes. Post-hoc Bonferroni analyses were then applied. *P* < 0.05 was considered as significant.

## Results

### Basal state in young and old in the cold

#### Basal state heart rate

**Fig. 1:**
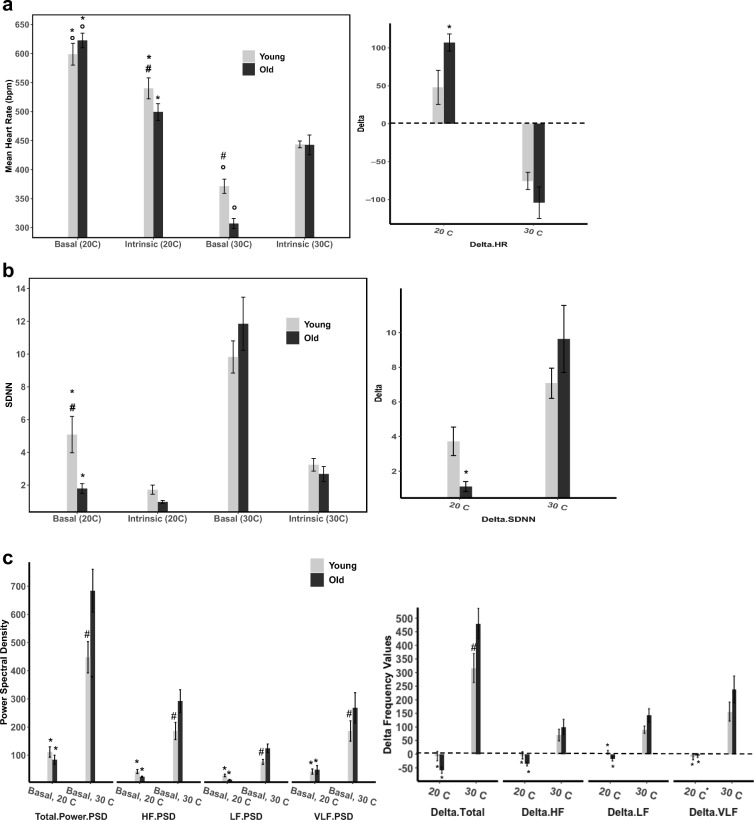

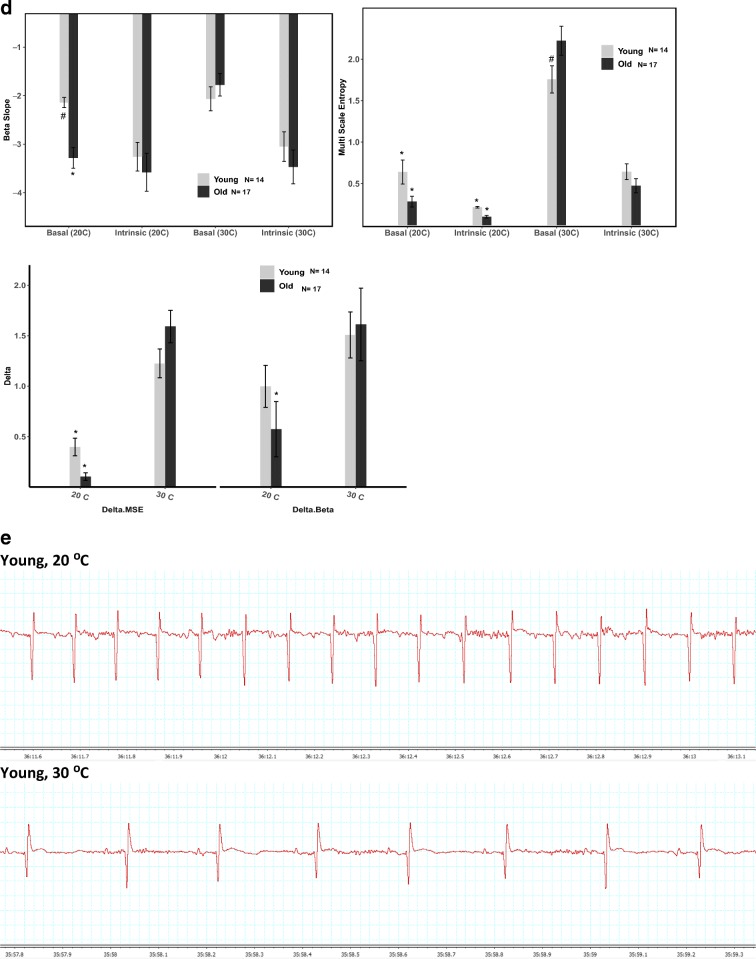
Basal-state, intrinsic-state, and Δ (basal state- intrinsic state) comparison values at 20 °C and 30 °C in young and old mice of **A** mean heart rate (bpm), **B** time-domain parameter SDNN, **C** frequency-domain parameters categories (power spectral density), **D** non-linear domain parameters multi-scale entropy and Beta-slope, and **E** representative ECG tracings from young and old mice at 20 °C and 30 °C, respectively. Data is reported as means with standard error bars. Linear mixed effects models were used to examine age, temperature, and drug effects and interactions between effects while accounting for uneven group sizes and repeated measures

At LT (20 °C) basal HR (BHR) did not significantly differ between young and old mice (Table 1, Fig. 1A). At TN (30 °C), both young and old mice had a substantially lower heart rate than when at LT (Table 1, Fig. 1A).

**Table 1:** Mean HR (bpm) values of young and old mice at 20 °C and 30 °C under both basal and intrinsic conditions. A *Δ* denotes a basal state-intrinsic state comparison at 20 °C or 30 °C within each age group. Data is reported as means (standard error). Linear mixed effects models were used to examine age, temperature, and drug effects and interactions between effects while accounting for uneven group sizes and repeated measures. See supplemental tables for significant comparisons between basal vs intrinsic states

Heart rate								
Parameter	Young				Old			
	20 °C		30 °C		20 °C		30 °C	
	Basal (*N* = 7)	Intrinsic (*N* = 7)	Basal (*N* = 13)	Intrinsic (*N* = 13)	Basal (*N* = 17)	Intrinsic (*N* = 17)	Basal (*N* = 13)	Intrinsic (*N* = 15)
Mean HR (bpm)	598* (19)	539*^#^ (18)	371^**#**^ (12)	443 (6)	622*(13)	499* (14)	307 (9)	442 (17)
Mean HR *Δ* (Basal-intrinsic)	47.91 (22.56)		− 75.39 (11.44)		109.46 (10.73)*		− 104.05 (20.71)	

*p < 0.05 vs 30 **°**C

^#^*p* < 0.05 vs. old mice

#### Basal state heart rate variability

##### Time-domain parameters

**Fig. 2:**
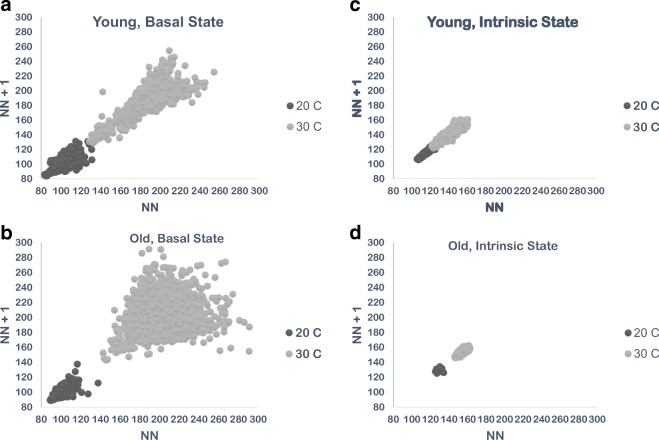
Poincare plots, in which the interbeat interval (NN) is plotted against the subsequent interbeat interval (NN+1) in a representative young and old mouse under basal conditions or after administration of a double-autonomic blockade

In old mice at LT, basal time-domain HRV was particularly reduced compared to that in young mice: the standard deviation of the NN interval (SDNN) was approximately 65% lower in old than in young mice (Table 2, Fig. 1B), and the coefficient of variation (CV), which adjusts the SDNN for the mean NN, was also about 65% lower in old than in young mice. The prominent age-associated differences in the time domain HRV are visualized in Poincaré plots, which portray the coordinates of a given inter-beat interval (NN) and the subsequent inter-beat interval (NN+1) (Fig. 2A and B). The coordinate points within Poincaré plots are tightly clustered when HRV is lower and more spread when HRV is higher.

**Table 2:** Time-domain parameter in young and older mice at 20 **°**C and 30 **°**C under both basal and intrinsic conditions. *Δ* denotes a basal state-intrinsic state comparison at 20 °C or 30 °C within each age group. Data is reported as means (standard error). Linear mixed effects models were used to examine age, temperature, and drug effects and interactions between effects while accounting for uneven group sizes and repeated measures. * signifies *p* < 0.05 when comparing a 20 **°**C state vs 30 **°**C state. # signifies *p* < 0.05 when comparing a young animal vs old animal. See supplemental tables for significant comparisons between basal vs intrinsic states

HRV in the Time Domain								
Parameter	Young				Old			
	20 °C		30 °C		20 °C		30 °C	
	Basal (N = 7)	Intrinsic (N = 7)	Basal (N = 14)	Intrinsic (N = 14)	Basal (N = 16)	Intrinsic (N = 15)	Basal (N = 15)	Intrinsic (N = 13)
SDNN	5.08#* (1.11**)**	1.72 (0.28)	9.82 (0.97)	3.23 (0.39)	1.78* (0.3)	0.97 (1.11)	11.85 (1.62)	2.67 (0.46)
***Δ****SDNN (Basal-Intrinsic)*	3.72 (0.83)		7.08 (0.87)		1.11 (0.29)*		9.64 (1.94)	
Coefficient of Variation (CV)	4.92**#** (1.02)	1.53 (0.23)	5.98 (0.46)	2.39 (0.28)	1.8* (0.28)	0.76 (0.06)	6.06 (0.8)	1.93 (0.28)
***Δ*** CV *(Basal-Intrinsic)*	3.71 (0.74)		3.73 (0.44)		1.06 (0.31)*		4.64 (1.63)

##### Frequency and non-linear domain parameters

**Fig. 3:**
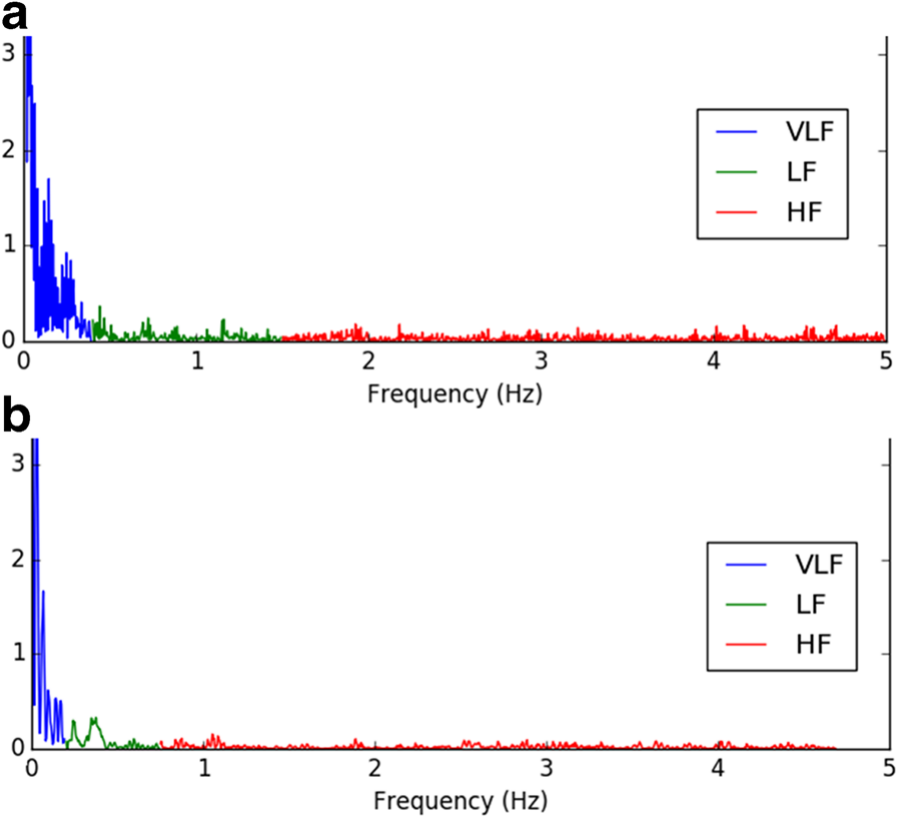
Power spectral density plots from a representative old animal at **A** 20 °C and **B** 30 °C. To account for marked differences in HR between ambient temperatures, different cut-off frequencies for different temperatures were used to categorize data (Behar et al. 2018): High-frequency power spectral density (HF.PSD) was defined as 1.50–5.0 Hz at 20 °C and 0.75–3.0 Hz at 30 °C. Low-frequency power spectral density (LF.PSD) was defined as 0.5–1.5 Hz at 20 °C and 0.2–0.75 Hz at 30 °C. Very low frequency power spectral density (VLF.PSD) was defined as 0–0.5 Hz at 20 °C and 0 to 0.2 Hz at 30 °C

An example of a power spectrum of a representative old mouse at LT is illustrated in Fig. 3A. At LT, all frequency domain power spectrum HRV parameters were lower in old vs young mice, indicating more coherency and less complexity in the heart rhythm of old vs young mice. These basal-state age deficits at LT, however, did not reach statistical significance (Table 3, Fig. 1C & D). Slope coefficient (*β*) of the non-linear domain power law function (log power spectrum density vs log frequency) increases (becomes more negative) as sympathetic input decreases (Yaniv et al. 2014). At LT, *β* of old mice (− 3.28 ± 0.21) was lower than that of young mice (− 2.14 ± 0.1, *p* < 0.06) (Table 4, Fig. 1D). The Hurst exponent reveals the extent of self-similarity in the non-linear domain of a time-series of NN intervals (Kale and Butar 2005): a Hurst exponent of 0.5 indicates a lack of autocorrelation among time intervals within an ECG time series, while a Hurst exponent of 0.5–1.0 indicates that a given interval predicts the next interval (Kale and Butar 2005). The basal-state Hurst exponent of old mice was slightly but significantly higher than that of young mice (0.79 ± 0.01 vs. 0.75 ± 0.01, *p* < 0.02) (Table 4, Fig. 1D).

**Table 3:** Frequency domain parameter in young and older mice at 20 **°**C and 30 **°**C under both basal and intrinsic conditions. *Δ* denotes a basal state-intrinsic state comparison at 20 °C or 30 °C within each age group. Data is reported as means (standard error). Linear mixed effects models were used to examine age, temperature, and drug effects and interactions between effects while accounting for uneven group sizes and repeated measures. * signifies *p* < 0.05 when comparing a 20 **°**C state vs 30 **°**C state. # signifies *p* < 0.05 when comparing a young animal vs old animal. See supplemental tables for significant comparisons between basal vs intrinsic states

HRV in the frequency domain								
Parameter	Young				Old			
	20 °C		30 °C		20 °C		30 °C	
	Basal (*N* = 7)	Intrinsic (*N* = 7)	Basal (*N* = 14)	Intrinsic (*N* = 14)	Basal (*N* = 16)	Intrinsic (*N* = 15)	Basal (*N* = 15)	Intrinsic (*N* = 13)
Total power PSD	95.94* (11.7)	126.39 (22.26)	413.07**#** (47.09)	200.4 (12.4)	63.73* (6.04)	128.86 (18.62)	683.89 (76.32)	230.8 (46.5)
*Δ* Total power PSD (basal-intrinsic)	− 7.04 (17.03)*		316. 53 (52.87)#		− 58.15 (11.86)*		480.28 (56.02)	
HF PSD	41.69* (7.21)	34.29 (3.58)	185.95**#** (30.29)	71.35 (7.44)	23.43* (1.68)	56.38 (9.42)	292 (40.29)	114.25 (29.28)
*Δ* HF PSD (basal-intrinsic)	− 4.52 (13.32)		70.05 (21.29)		− 35.82 (6.67) *		99.95 (27.94)	
LF PSD	27.91* (4.74)	21.19 (5.49)	76.01**#** (8.95)	16.43 (2.05)	12.39* (1.33)	23.82 (4.78)	123.98 (15.77)	17.26 (3.51)
*Δ* LF PSD (basal-intrinsic)	6.80 (7.03)*		90.01 (13.18)		− 17.12 (7.49)*		142.16 (24.90)	
VLF PSD	33.44* (5.34)	38.82 (11.37)	152.76**#** (15.54)	83.99 (14.93)	29.21* (4.9)	54.35 (12)	267.9 (54.1)	91.09 (30.56)
*Δ* VLF PSD (basal-intrinsic)	− 9.32 (11.14)*		156.45 (34.85)		− 5.21 ( 6.75)*		238.17 (49.50)

**Table 4:** Non-linear domain heart rate variability parameter in young and older mice at 20 **°**C and 30 **°**C under both basal and intrinsic conditions. *Δ* denotes a basal state-intrinsic state comparison at 20 °C or 30 °C within each age group. Data is reported as means (standard error). Linear mixed effects models were used to examine age, temperature, and drug effects and interactions between effects while accounting for uneven group sizes and repeated measures. * signifies *p* < 0.05 when comparing a 20 **°**C state vs 30 **°**C state. # signifies *p* < 0.05 when comparing a young animal vs old animal. See supplemental tables for significant comparisons between basal vs intrinsic states

HRV in the non-linear domain								
Parameter	Young				Old			
	20 °C		30 °C		20 °C		30 °C	
	Basal (*N* = 7)	Intrinsic (*N* = 7)	Basal (*N* = 14)	Intrinsic (*N* = 14)	Basal (*N* = 16)	Intrinsic (*N* = 15)	Basal (*N* = 15)	Intrinsic (*N* = 13)
Beta slope	− 2.14**#** (0.1)	− 3.26 (0.3)	− 2.07 (0.25)	− 3.05 (0.3)	− 3.28* (0.21)	− 3.58 (0.9)	− 1.78 (0.23)	− 3.47 (0.35)
*Δ* Beta slope (basal-intrinsic)	1.00 (0.21)		1.51 (0.23)		0.57 (0.27) *		1.61 (0.36)	
MSE	0.64* (0.14)	0.21* (0.01)	1.76**#** (0.16)	0.64 (0.1)	0.28* (0.07)	0.1* (0.01)	2.22 (0.17)	0.47 (0.09)
*Δ* MSE (basal-intrinsic)	0.40 (0.09)*		1.22 (0.14)		0.10 (0.04)*		1.59 (0.16)	
DFA	0.98 (0.04)	1.15 (0.03)	0.84 (0.07)	1.2 (0.02)	1.09* (0.05)	1.26 (0.05)	0.85 (0.06)	1.15 (0.03)
*Δ* DFA (basal-intrinsic)	47.91 (22.56) *		− 75.39 (11.44)		109.46 (10.73) *		− 104.05 (20.71)	
Hurst	0.75**#** (0.01)	0.78 (0.01)	0.74 (0.02)	0.78 (0.01)	0.79* (0.01**)**	0.79 (0.01)	0.73 (0.01)	0.81 (0.01)
*Δ* Hurst (basal-intrinsic)	− 0.04 (0.02)		− 0.06 (0.02)		0.01 (0.01)*		− 0.06 (0.02)	

### Complexity emerges at thermoneutrality

#### Basal state heart rate

**Fig. 4:**
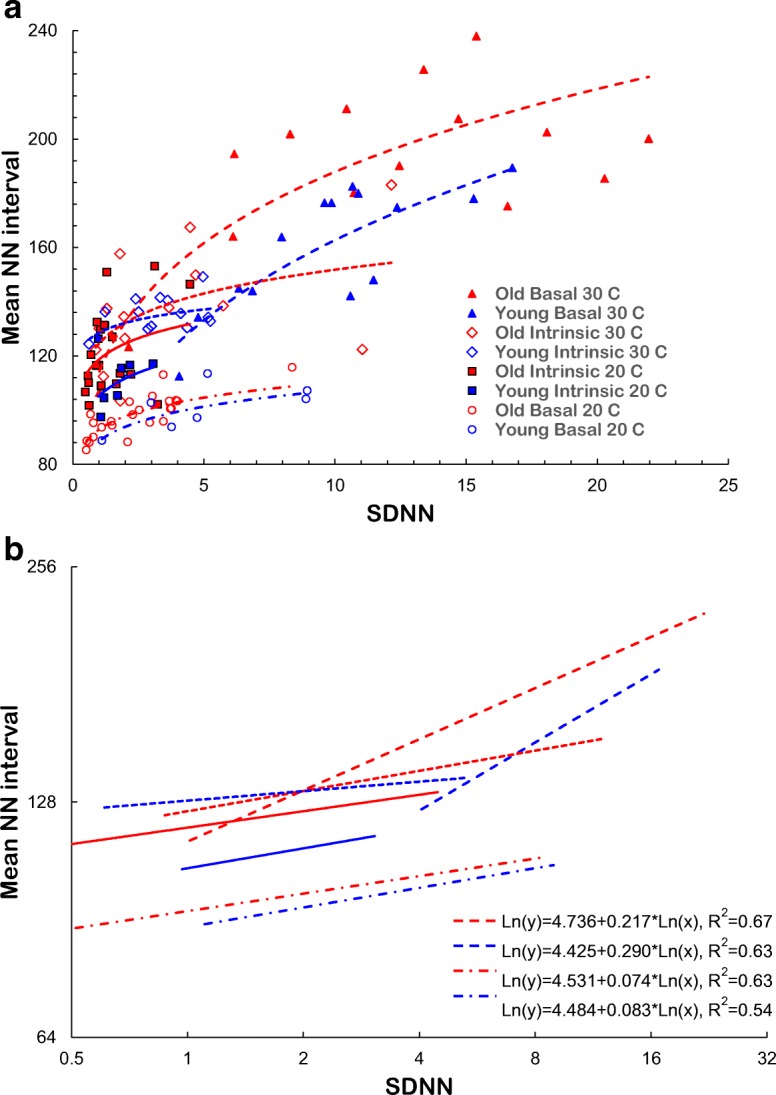
Age and ambient temperature dependence of the relationship of Mean NN to SDNN. **A** The NN vs SDNN relationship in both age groups in the basal and intrinsic states during acclimation at 20 °C and 30 °C. **B** Power-law functions (lnNN-lnSDNN) for the data in Panel A

Compared to LT, BHR of young mice at TN decreased 38% (*p* < 0.0001); that of old mice decreased by 51% (*p* < 0.0001), leading to a lower BHR in old vs young mice at TN (*p* < 0.002) (Table 1, Fig. 1A). Acclimation at the neutral metabolic ambient temperature (TN, 30 °C) shifted the basal heart rhythm from the coherent pattern observed at LT (20 °C) to a more complex pattern in both age groups (Tables 2, 3, and 4). The increased complexity of the heart rhythm at TN is clearly visualized in Poincaré plots at the two temperatures (Fig. 2A and B). Complete patterns of complexity or coherence within the heart rhythm become manifest in plots of Mean NN vs. SDNN (Monfredi et al. 2014). A change in the ambient temperature shifted the well-described shape of the non-linear relationship of Mean NN to SDNN in the basal state (Fig. 4A). As the Mean NN interval time shortens, the SDNN decreases. Figure 4B illustrates ln-ln (power-law) plots of the Mean NN-SDNN relationship in Fig. 4A to determine whether ambient temperature affects their power-law behavior (Yaniv et al. 2014).

#### Basal state heart rate variability

##### Time-domain parameters

Basal time-domain HRV increased in both age groups of mice after acclimation to TN (Table 2, Fig. 1B): all time-domain HRV parameters at TN were increased compared to the those at LT (Table 2, Fig. 1B): while the temperature difference in all HRV parameters were statistically significant in old mice, in young mice, only select parameters reached statistical significance.

Time domain HRV parameters of old mice were much more affected by temperature than those in young mice by acclimation to TN. Age differences in HRV observed at LT are reduced or abolished at TN. Specifically, basal SDNN of old mice increased over fivefold between LT and TN (*p* < 0.0001), while that of young mice increased by 93% (*p* < 0.0005), reducing the difference between the two age groups that was marked at LT (*p* = 0.06) (Table 2, Fig. 1B). The CV of NN intervals of old mice at TN increased over twofold higher from that at LT (*p* < 0.0002), but young mice increased by only 22% (*p* < 0.2), abolishing the age difference in CV that was striking at LT (*p* = 0.8352) (Table 2, Fig. 1B).

##### Frequency and non-linear domain parameters

Significant increases in high frequency (HF.PSD), low frequency (LF.PSD), and very low frequency power (VLF.PSD) components of the basal heart rhythm occurred in both age groups when the ambient temperature was warmed from LT to TN (Table 3, Fig. 1C). A power spectrum of a representative old mouse at LT is supplied in Fig. 3B. Compared to LT, basal HRV non-linear domain parameters of old mice were significantly higher at TN (Table 4). In young mice, the non-linear domain HRV pattern was also increased vs that at LT, but as in LT, a statistically significant temperature dependence in young mice was only manifested in some parameters (Table 4, Fig. 1D).

Age differences in non-linear domain parameters were also substantially affected by the ambient temperature. In old mice, *β* showed a marked increase (more negative by 46%) at TN vs LT (*p* < 0.0003), but in young mice *β* increased very slightly (3%) between LT and TN (*p* < 0.8), rendering the age difference observed in B at LT non-significant at TN (*p* < 0.6) (Table 4, Fig. 1D). Old mice also had a large (sixfold) increase in MSE at TN vs LT, and although MSE in young mice increased almost twofold, this increase was significantly lower than that of the old mice (*p* < 0.005) (Table 4, Fig. 1D).

### Intrinsic state HR and HRV in the young and old in the cold

Double autonomic blockade was administered to reduce ANS input in order to discern intrinsic heart rate (IHR). In contrast to basal HR at LT, IHR of old mice was reduced compared to that of young mice (*p* < 0.05). There were no significant age differences in intrinsic HRV at LT, suggesting that many of the age disparities observed in the basal state at LT (Tables 2, 3, and 4) are largely due to age differences in autonomic input or to SAN cell response to that input.

### Intrinsic HR & HRV after warming things up

When acclimated at TN, IHR became significantly reduced in both young and old mice compared to LT; IHR of young mice decreased by 18% (*p* < 0.0001) and IHR of old mice decreased by 11% (*p* < 0.0003) (Table 1, Fig. 1A). In contrast to LT, however, IHR was nearly identical in young and old mice at the warmer thermoneutral temperature. Intrinsic heart rhythm complexity at TN increased in both age groups compared to LT, but these trends did not reach statistical significance, except for Multiscale Entropy (MSE) which was significantly higher in both age groups at TN than at LT (Tables 2, 3, and 4, Fig. 1D). These minimal effects can be visualized using Poincaré plots (Fig. 2C and D) and the Mean NN-SDNN relationship (Fig. 4A & B).

### Ambient temperature dependence of HR and HRV response to dual autonomic blockade

We reasoned that the difference between BHR and IHR at each given temperature, i.e., the *Δ* HR and *Δ* HRV, reflects a rough index of autonomic influence on HR and HRV. In this regard, the effects of double autonomic blockade on heart rate were dependent on the temperature: at LT, double autonomic blockade reduced heart rate in both age groups, but the effect of double autonomic blockade was greater in old than in young, rendering a lower IHR in old vs young at LT (Table 1, Fig. 1A). But at TN, heart rate increased following the double autonomic blockade, as it does in humans (Table 1, Fig. 1A). Specifically, the Δ HR of old mice at LT was + 109.46 (10.73) bpm, but significantly decreased to − 104 (10.72) at TN (*p* < 0.0001) (Table 1, Fig. 1A), while Δ HR of young mice at LT was + 47.91 (22.56) and decreased to − 75.39 (11.44) at TN (*p* = 0.32) (Table 1, Fig. 1A). This increase in basal time-domain HRV is also demonstrated in the larger *Δ* values (basal-intrinsic) of SDNN and CV, but again this increase was only significant in old mice (Table 3, Fig. 1B). Age or temperature differences in frequency and non-linear domains were also observed in the larger *Δ* values (basal-intrinsic) of *β*-slope and Multiscale Entropy (Tables 3 and 4, Fig. 1C and D).

### Core body temperature

Ambient temperature did not affect basal-state core body temperatures (Table 5). In the intrinsic state, however, core body temperature of young mice was slightly lower at LT compared to TN (*p* < 0.05) (Table 5). Additionally, young mice in both the basal and intrinsic states had a higher core body temperature at LT than old mice (*p* < 0.05) (Table 5).

**Table 5:** Core body temperature of young and older mice extracted from telemetry devices during basal state recordings at 20 °C and 30 °C. Data is reported as means (standard error). Linear mixed effects models were used to examine age, temperature, and drug effects and interactions between effects while accounting for uneven group sizes and repeated measures. A * signifies *p* < 0.05 when comparing a 20 **°**C state vs 30 **°**C state. A # signifies *p* < 0.05 when comparing a young animal vs old animal. See supplemental tables for significant comparisons between basal vs intrinsic states

Core body temperatures								
	Basal				Intrinsic			
	Young (*n* = 9)		Old (*n* = 16)		Young (*n* = 9)		Old (*n* = 16)	
Temperature (°C)	20 °C	30 °C	20 °C	30 °C	20 °C	30 °C	20 °C	30 °C
	36.48 (0.17)	36.97# (0.26)	36.05 (0.21)	35.85 (0.17)	35.74* (0.31)	36.75# (0.31)	35.89 (0.21)	35.25 (0.14)

*p < 0.05 vs 30 C

^#^*p* < 0.05 vs. old mice

## Discussion

### Thermoneutrality rescues the age-associated decline in HRV

The major finding of our study is that the age-associated decline in heart rhythm complexity is abolished when old animals are acclimated to their metabolic thermoneutral zone.

When mice were acclimated to 20 °C, the old mouse group had lower heart rate variability than the young mice across a wide array of parameters, confirming previous observations (Liu et al. 2014; Yaniv et al. 2014). Our study is the first to our knowledge, to provide in-depth analysis of mouse HRV response to ambient temperature. Our results indicate that at LT, old mice had lower HRV than young mice across all three HRV domains (Tables 2, 3, and 4). But, when both age groups were acclimated to their TN, age differences between the two groups were reduced or ameliorated, and old mice, in fact, actually manifested an increased HRV pattern at TN compared to young mice. This is commensurate with the lower HR of old vs young mice at TN (Table 1, Fig. 1) and the well-known inverse relationship between HR and HRV (Monfredi et al. 2014).

The effect of thermoneutrality on prominent age differences in HRV in the time domain was also observed in the frequency domain analysis. At LT, there were trends for HRV in the frequency-domain in the young mice to exceed those of old mice, yet at TN, old mice had significantly higher values than young mice in all frequency-domain parameters (Table 3). In other terms, the age difference in the frequency HRV domain at LT was reversed at TN. And age differences in HRV parameters at LT were either abolished or substantially recued at TN.

### Excessive sympathetic drive to mouse heart at the standard ambient laboratory temperature

At standard laboratory temperatures (LT) (~ 20–22 °C) mice, by default, use an overdrive of sympathetic impulses from autonomic brain stem nuclei to effect non-shivering thermogenesis vis stimulation of brown adipose tissue in order to maintain a normal core body temperature (Himms-Hagan 1985; Kawate et al. 1994; Swoap et al. 2008, 2004). Values of non-linear domain HRV parameters that describe variation in fractal like systems that possess complex and chaotic variability at 20 °C (Fig. 1D, Table 4) confirm the loss of complexity in cold-stressed mice observed in the time and frequency domain analyses. This hyper-adrenergic state has distorted interpretation of mouse heart cardiovascular regulation and autonomic nervous system input. Increased sympathetic stimulation at LT causes a chronically elevated heart rate and a reduction in heart rate variability. Low heart rate variability has been associated with poor clinical outcomes, including increased mortality rates in post-MI patients (Bigger et al. 1992a, b, 1993; Thayer et al. 2010). This coherent rhythm at 20 °C resembles that of several conditions associated with high-sympathetic drive, including PTSD, panic disorder, and generalized anxiety disorder (Cohen et al. 2000). Individuals with low HRV have also been found to have delayed recovery in response to stress, suggesting a less robust homeostatic response (Thayer et al. 2012). Thus, chronic cold stress–induced sympathetic drive might accelerate the deterioration in heart health in aged mice.

### Dominant vagal tone and increased heart rate variability emerge at thermoneutrality

When mice are acclimated to their thermoneutral zone, sympathetic input to the heart is greatly reduced, and a parasympathetic HRV pattern emerges. Both time and all frequency domain HRV parameters increased in both age groups when mice were acclimated to 30 °C, indicative of a large vagal component of mouse heart rate regulation at 30 °C that was overridden at 20 °C by a high sympathetic tone. Reduced VLF power, the strongest predictor of adverse clinical outcomes (Bigger et al. 1992a, b, 1993; Kleiger et al. 1987; La Rovere et al. 1998), was markedly increased in mice of both ages at 30 °C, suggesting better cardiovascular health status for mice at TN.

### Thermoneutrality reverses HR response to dual autonomic blockade

Administration of a dual autonomic blockade is intended to diminish input from both the sympathetic and parasympathetic arms of the autonomic nervous system, revealing intrinsic SAN cell activity. Our results at LT confirmed previous results that HR is reduced in mice by the administration of a dual autonomic blockade (Yaniv et al. 2016), suggesting that under basal conditions at LT, the murine heart is sympathetically-controlled. When we administered a dual autonomic blockade at TN, however, HR increased, confirming the findings of Swoap et al. that thermoneutrality reveals a strong parasympathetic component of murine heart regulation. This is especially important as human HR is parasympathetically controlled (Billman 2011). In the context of a translational model that recapitulates human physiology, it is vital that mice be housed under thermoneutral conditions to allow for normal autonomic regulation of HR and HRV.

### Mice maintained basal-state core body temperature despite cold stress

Under basal conditions, both young and old mice were able to maintain standard core body temperatures (36–37 °C) at both LT and TN. This is further evidence there must be a mechanism ensuring core body temperature homeostasis despite changes in ambient temperature. This mechanism has previously been identified as sympathetic stimulation of BAT to induce non-shivering thermogenesis (Himms-Hagan 1985; Kawate et al. 1994; Swoap et al. 2008, 2004). After administration of dual autonomic blockade at LT, which diminishes sympathetic activity, young mice were unable to maintain core body temperature in the intrinsic state at LT. Old mice, however, in both the absence or presence of autonomic blockade had a slightly but significantly lower core body temperature than young mice at TN, which confirms previous findings of a decrease in core body temperature with age (Sanchez-Alavez et al. 2011). Old mice, however, did not experience a decrease in core body temperature even in the intrinsic state at LT, suggesting that chronic exposure to LT might cause additional changes in core body temperature regulation.

## Summary

Reductions in HR, loss of complexity, and an increase in coherency of heart rhythm in mice as they age (Yaniv et al. 2016; Larson et al. 2013) may largely be attributable to the ambient LT (20–22 °C) at which the mice were acclimated. Importantly, our results are the first to not only provide in-depth analysis of mouse HRV response to ambient temperature but also show for the first time that acclimation to a thermoneutral ambient temperature (30 °C) reverses age-associated declines in HR and HRV at standard laboratory ambient temperature (20 °C).

Mice are commonly used as a translational model due to their short lifespan, quick reproduction time, ease of keep, and transgenic availability. Translational models, though, aim to mimic human physiology as closely as possible. Under the chronic cold stress of acclimation to room temperature, mice operate under high sympathetic drive in order to maintain a normal core temperature. This distorts perspective on autonomic nervous system regulation of the structure and function of the cardiovascular system, particularly HR, HRV, and their autonomic regulation. A true understanding and elucidation of changes in the brain-heart-brain cross-talk that accompany advancing age, requires that mice be acclimated to their metabolic thermoneutral environment.

## Electronic supplementary material

ESM 1 (DOCX 17 kb)

## Time-domain parameters

NN interval: Time between two successive ECG R peaks
SDNN: Standard deviation of all NN intervals
CV: Coefficient of variation (SDNN divided by mean of all NN intervals)
Range NN: Difference between the longest NN interval and shortest NN interval
Median NN: Median length of NN interval

## Frequency domain parameters

Total power PSD: Total power spectral density
VLF PSD: Very low frequency power spectral density
LF PSD: Low frequency power spectral density
HF PSD: High frequency power spectral density

## Non-linear domain parameters

Beta slope: Slope of the linear function after the exponential relationship of the power spectral density to the power spectrum frequency transformed via log-log scale
Multiscale entropy (MSE): Quantifies degree of randomness
Detrended fluctuation analysis (DFA): Describes the correlations between successive NN intervals
Hurst exponent: Quantifies multifractal behavior in a time-series and describes self-similarity in a system
