# Supplementary material for: Acclimation to a thermoneutral environment abolishes age-associated alterations in heart rate and heart rate variability in conscious, unrestrained mice
Source: GeroScience. 2019 Nov 27;42(1):217–32. doi: 10.1007/s11357-019-00126-7 (PMC7031176; doi:10.1007/s11357-019-00126-7)
Supplement: Supplementary file 1 — (DOCX 17 kb) [file 11357_2019_126_MOESM1_ESM.docx]

**°P < 0.05 vs intrinsic condition.**

| **Heart Rate** | | | | | | | | |
| --- | --- | --- | --- | --- | --- | --- | --- | --- |
| **Parameter** | **Young** | | | | **Old** | | | |
|  | **20°C** | | **30°C** | | **20°C** | | **30°C** | |
|  | **Basal**  (N=7) | **Intrinsic**  (N=7) | **Basal**  (N=13) | **Intrinsic**  (N=13) | **Basal** (N=17) | **Intrinsic** (N=17) | **Basal**  (N=13) | **Intrinsic**  (N=15) |
| Mean HR (bpm) | 598**°** (19) | 539 (18) | 371**°** (12) | 443  (6) | 622**°** (13) | 499  (14) | 307 **°**  (9) | 442  (17) |

| **Time Domain Parameters** | | | | | | | | |
| --- | --- | --- | --- | --- | --- | --- | --- | --- |
|  | Basal State | | | | Intrinsic State | | | |
|  | Young | | Old | | Young | | Old | |
|  | 20**°**C | 30**°**C | 20**°**C | 30**°**C | 20**°**C | 30**°**C | 20**°**C | 30**°**C |
| SDNN | 5.08 (1.11)**°** | 9.82 (0.97)**°** | 1.78 (0.3) | 11.85 (1.62)**°** | 1.72 (0.28) | 3.23 (0.39) | 0.966 (1.11) | 2.67 (0.46) |
| CV | 4.92 (1.02)**°** | 5.98 (0.46)**°** | 1.8 (0.28)**°** | 6.06 (0.8)**°** | 1.53 (0.23) | 2.39 (0.28) | 0.76 (0.06) | 1.93 (0.28) |

**Frequency Domain Parameters**

|  | Basal State | | | | Intrinsic State | | | |
| --- | --- | --- | --- | --- | --- | --- | --- | --- |
|  | Young | | Old | | Young | | Old | |
|  | 20**°**C | 30**°**C | 20**°**C | 30**°**C | 20**°**C | 30**°**C | 20**°**C | 30**°**C |
| VLF PSD | 33.44 (5.34) | 152.76 (15.54) | 29.21 (4.9) | 267.9 (54.1)**°** | 38.82 (11.37) | 83.99 (14.93) | 54.35 (12) | 91.09 (30.56) |
| LF PSD | 27.91 (4.74) | 76.01 (8.95)**°** | 12.39 (1.33) | 123.98 (15.77)**°** | 21.19 (5.49) | 16.43 (2.05) | 23.82 (4.78) | 17.26 (3.51) |
| HF PSD | 41.69 (7.21) | 185.95 (30.29) **°** | 23.43 (1.68) | 292 (40.29)**°** | 34.29 (3.58) | 71.35 (7.44) | 56.38 (9.42) | 114.25 (29.28) |
| Total Power PSD | 95.94 (11.7) | 413.07 (47.09)**°** | 63.73 (6.04) | 683.89 (76.32)**°** | 126.39 (22.26) | 200.4 (12.4) | 128.86 (18.62) | 230.8 (46.5) |

**Nonlinear Domain Parameters**

|  | Basal State | | | | Intrinsic State | | | |
| --- | --- | --- | --- | --- | --- | --- | --- | --- |
|  | Young | | Old | | Young | | Old | |
|  | 20**°**C | 30**°**C | 20**°**C | 30**°**C | 20**°**C | 30**°**C | 20**°**C | 30**°**C |
| Beta Slope | -2.14 (0.1) | -2.07 (0.25)**°** | -3.28 (0.21) | -1.78 (0.23)**°** | -3.26 (0.3) | 3.05 (0.3) | -3.58 (0.9) | -3.47 (0.35) |
| MSE | 0.64 (0.14)**°** | 1.76 (0.16)**°** | 0.28 (0.07) | 2.22 (0.17)**°** | 0.21 (0.01) | 0.64 (0.1) | 0.1 (0.01) | 0.47 (0.09) |
| DFA | 0.98 (0.04) | 0.84 (0.07)**°** | 1.09 (0.05)**°** | 0.85 (0.06)**°** | 1.15 (0.03) | 1.2 (0.02) | 1.26 (0.05) | 1.15 (0.03) |
| Hurst | 0.75 (0.01) | 0.74 (0.02)**°** | 0.79 (0.01) | 0.73 (0.01)**°** | 0.78 (0.01) | 0.78 (0.01) | 0.79 (0.01) | 0.81 (0.01) |
